# Effect of Human Attachment Style on Horse Behaviour and Physiology during Equine-Assisted Activities–A Pilot Study

**DOI:** 10.3390/ani10071156

**Published:** 2020-07-08

**Authors:** Aitor Arrazola, Katrina Merkies

**Affiliations:** 1 Department of Animal Biosciences, University of Guelph, Guelph, ON N1G 2W1, Canada; aarrazol@uoguelph.ca; 2 Campbell Centre for the Study of Animal Welfare, University of Guelph, Guelph, ON N1G 2W1, Canada

**Keywords:** equine welfare, therapy horses, physiological stress, behavioural stress, attachment style, heart rate, affiliative behaviour, insecure adolescent, animal–human interaction, bonding

## Abstract

**Simple Summary:**

Human–horse interaction, such as attachment and bonding behavior, is particularly important during repeated equine-assisted activities (EAA). This bond needs to be reliable, positive, and reciprocal to create a secure human–horse attachment and achieve improvements in EAA participants. This study aims to determine the effect of the attachment style (AS) of at-risk adolescents on the physiology and behavior of therapy horses during a 10-week EAA. Adolescents completed a questionnaire to determine their AS before starting the program. Horse response during the EAA was recorded, looking at horse heart rate and behaviour (the occurrence of affiliative and avoidance behaviours). Adolescents’ AS affected horse affiliative behaviour during grooming, and horse heart rate and avoidance behaviour during riding. The results indicate that horse welfare was not threatened during the EAA with at-risk adolescents. Over time, therapy horses showed overall more affiliative behaviour and less variability in their stress response (heart rate and avoidance behaviours) toward insecure AS adolescents during grooming and riding, respectively. These results suggest that insecure AS can positively impact horse response during EAA relative to secure AS in humans. However, the implications of human AS on the functioning of horse–human interaction and its mechanisms are yet unknown.

**Abstract:**

Equine-assisted activities (EAA) for human well-being and health rely on human–horse interactions for therapeutic effect. At-risk participants with mental and emotional difficulties can show poor social skills and functioning relationships, potentially leading to unsuccessful human–horse interaction in EAA. This study addresses the effect of the attachment style (AS) of at-risk adolescents on horse physiology and behaviour during an equine-facilitated learning (EFL) program. Thirty-three adolescents participated in a 10-week EFL program with nine therapy horses (the same therapy horse per adolescent throughout the program). Adolescent AS was categorized into secure (n = 7), preoccupied (n = 11), dismissing (n = 1), or fearful (n = 12) using an Experiences in Close Relationships – Relationship Structure questionnaire. Horse heart rate (HR) and behaviour (affiliative and avoidance behaviours) in response to adolescents were recorded during grooming and riding. Over time, horses with fearful AS adolescents showed consistently more affiliative behaviours compared to those with preoccupied AS adolescents during grooming, and more constant HR and avoidance behaviours compared to those with secure AS adolescents during riding. These results suggest that a more predictable and less stressful physiological and behavioural response of therapy horses toward participants in EAA with emotional and behavioural difficulties can be mediated by a human insecure attachment style.

## 1. Introduction

Equine-assisted activities (EAA) refers to a variety of approaches, whereby guided interactions between humans and equids facilitate some positive effects on human function and well-being [1,2]. This may incorporate physical or psychological therapy, or simply facilitate awareness of personal skills. Indeed, EAA is a recommended approach to alleviate mental and physical difficulties and enhance the health and well-being of at-risk humans [1,3]. Equine-assisted activities, such as equine-assisted therapy (EAT) and equine-facilitated learning (EFL), are becoming a preferred treatment plan for mental and emotional disorders [3,4], and the success of EAT and EFL relies on the bond between human and horse [1]. This bond needs to be reliable, positive, close, and reciprocal to create a secure attachment between human and horse [2,5,6]. Evidence of a bond, as viewed from the human perspective, universally includes the horse approaching them, vocally greeting them, trusting them in a dangerous situation, taking care of them, and initiating physical contact [7]. Equine research has indicated that horses can show proximity- and contact-seeking behaviours [8,9,10] and separation distress toward humans [9,11], and humans can be a secure base for exploration [12,13] and a safe haven to return to when horses are distressed [14]. These traits are crucial signs of attachment-like behaviour from horses toward humans [6], and this predisposing bonding behaviour is desirable in therapy animals [1,5]. Horses can provide sensory, emotional, and cognitive stimulation, particularly important for at-risk adolescents involved in EAA [2]. Thus, horses are viewed as a co-therapist in EAA to improve the well-being and health of participants [5]. 

The adolescent–horse attachment relationship is suggested to be crucial in EAT and EFL programs, and functions as a primary mechanism for therapeutic improvement [2,4]. Mueller and McCullough [4] indicated that adolescents in EAT can create an immediate strong connection with horses, and adolescents self-reporting high bonding scores showed less post-traumatic stress symptoms at the end of a 10-week therapy program. The way in which humans interact with others and feel about their relationships varies broadly, resulting in a range of attachment styles (AS) [15]. Certainly, the AS of humans can affect their perception of their social world, influencing their social skills, emotional health, and mental well-being [15]. For example, humans with a secure AS are more likely to have good social skills, well-functioning relationships, and positive self-image, leading to low depression-like and distress-like symptoms. Secure and insecure AS can be determined by the degree of anxiety and avoidance in the way that humans behave in close relationships [15,16]. Highly anxious AS is associated with the need for continuous reinforcement and reassurance of their relationships due to wariness and feelings of abandonment [6,15]. Highly avoidant AS are associated with detachment and a lack of emotional investment into their relationships due to fearful feelings regarding emotional closeness, dependence on others, and opening-up to others [6,15]. Further, Zilcha-Mano et al. [17,18] determined that a human’s AS is mirrored in their relationship with animals. At-risk adolescents (e.g., those with insecure AS: dismissing, preoccupied, and fearful) are often enrolled in EAT and EFL programs because of problems forming and maintaining relationships, poor social skills, and mistrusting people [4,5,19]. The poor relationship functioning associated with insecure AS can weaken the human–animal relationship during EAA [20] and potentially compromise the therapeutic and educational effect of EAT and EFL, respectively. 

The quality of the interaction between adolescents and therapy horses in EAA has direct consequences on the behaviour and welfare of both [6,21]. For EAT and EFL programs to be successful, adolescents (either with secure or insecure AS) are expected to form an attachment relationship with their horse [4,5]. However, the horse’s response toward adolescents’ AS is unknown. Horses are particularly susceptible to interpreting specific actions of human body language as threatening or frightening [6], and safety concerns can be raised if horses show aggressive or avoidant behaviour in response to fear [21,22,23]. Therefore, the objective of this research was to assess the effect of the attachment style of at-risk adolescents on the horse physiology and behaviour during a typical EFL program.

## 2. Materials and Methods

A total of 33 at-risk adolescents participated in a 10-week equine-facilitated learning (EFL) program at Jewel View Farm in Orangeville (Ontario, Canada) from May 2019 to July 2019. The procedures in this study were approved by the Research Ethics Board at the University of Guelph for the use of human research subjects (REB 18-06-014) and by the Animal Care Committee at the University of Guelph (AUP3344) under the Canadian Council of Animal Care for the use of animals in teaching and research [24].

### 2.1. Animals

Nine therapy horses participated in the EFL program. All therapy horses were geldings of various breeds ranging from 9 to 30 years old. All but one horse had at least one year of experience as a therapy horse. From the researcher’s observations before daily adolescent arrival, horses were behaviorally (e.g., no signs of aggressiveness, arousal, attempts to escape, or distress) and physically healthy (e.g., no signs of injuries, sickness, poor gait or respiratory problems). All horses lived as a group outside in paddocks containing run-in shelters. Selected horses were brought into the barn at least one hour before adolescents arrived. During this time, horses were allotted to their respective stalls, fed hay ad libitum, and checked for any injuries. Horses were not used in more than one therapy session per day, and the average weekly number of adolescents (i.e., weekly workload) per therapy horse was 3.6, spread over two to three working days. However, the weekly workload ranged among horses from 1.6 to 6.4 adolescents per week. 

### 2.2. Humans

Adolescents (18 females, 12 males, and 3 non-binary gender) between 12 to 19 years old and at-risk for mental health and behavioural difficulties were recruited to participate in the EFL program from three different residential mental health agencies. Adolescents participated in the program consistently on the same weekday for 10 weeks. Most adolescents had not participated in riding before, and all were beginner riders. Adolescents from the same agency were paired together and assigned to the same therapy horse according to their weight and height. Adolescents remained with the same horse throughout the duration of the EFL program, and some horses were assigned to more than one pair of adolescents. The overall participation rate (i.e., at least 7 out of 10 sessions) in this EFL program was 75.8% (25 out of 33 adolescents).

### 2.3. Equine-Facilitated Learning (EFL)

Equine-facilitated learning is a type of EAA and refers to an experimental learning modality to improve the emotional and mental well-being of the client using horses guided by an instructor to achieve determined learning milestones [1,25]. In this research, the EFL program lasted for 10 weeks and consisted of weekly sessions with a grooming component, a riding component, and a debriefing component (as a group) in each session. During each session, the number of horses working simultaneously ranged from three to five therapy horses plus the instructor’s horse. The grooming component lasted for approximately 15 min, and paired adolescents interacted with their horse via grooming and tacking while in the stables. Adolescents rode individually in an indoor riding arena, with the first adolescent riding for approximately 30 min followed by the second adolescent. The order in which the adolescents rode was decided among themselves and noted by the researchers. The instructor and volunteers (one per therapy horse) assisted the adolescents, especially during the first sessions of the EFL program. During the grooming component, adolescents learned common management and handling practices in the first sessions (e.g., brushing) and more advanced ones in the last sessions (e.g., combing horse’s tail, and cleaning hooves). During the riding component, adolescents progressed from leading their horse on the ground in the first session, to mounting and being led by the volunteer in the second session, to riding on their own from the third session onward (increasing riding intensity and complexity over time). The last session was a demonstration day when adolescents showed their riding skills to an invited audience (e.g., family members). All arena work was done at the walk. Researchers recorded the horse response for each session in person before the adolescents’ arrival (i.e., baseline) and during the grooming and riding components (see below). The debriefing component was not part of this study due to the private and personal character of this component, and adolescents scarcely interacted with the horses at this time.

### 2.4. Data Collection

#### 2.4.1. Adolescent Data

**Figure 1:**
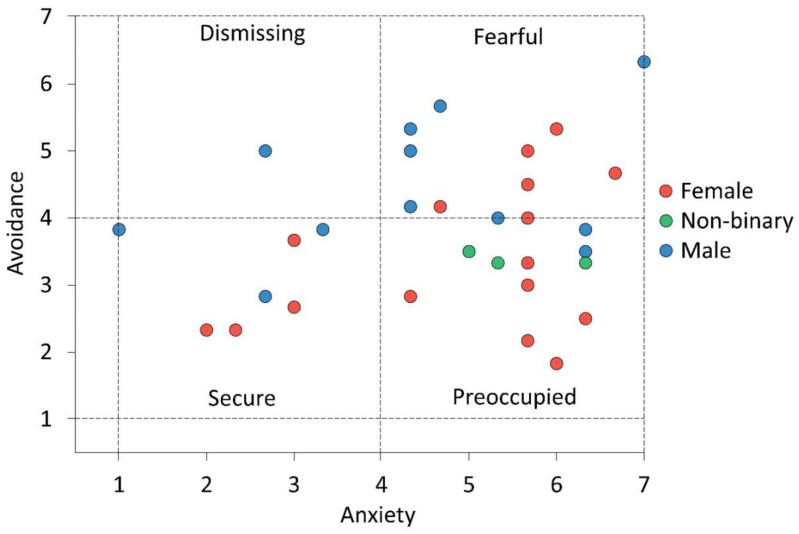
The attachment style (AS) of at-risk adolescents (n = 31) in this equine-facilitated learning (EFL) program based on their anxiety and avoidance scores. The gender of the adolescents is illustrated in red for female, green for non-binary, and blue for male. The adolescents’ AS (i.e., quadrants) was determined using an Experiences in Close Relationships-Relationship Structure (ECR-RS) questionnaire [15,16]. The ECR-RS questionnaire was developed to quantify the degree of anxiety and avoidance in their personal relationship with their mother, father, romantic partner, and best friends [16]. Therefore, this AS represents the average score of at-risk adolescents according to four types of close relationships with their parents, friends, and partners.

Adolescents completed the Experiences in Close Relationships-Relationship Structure (ECR-RS) questionnaire (developed by Fraley et al. [16]) prior to starting the EFL program to determine their AS. Fraley and colleagues [16] aimed to develop a practical and relatively short tool to assess the AS of participants toward specific and multiple close relationships, while allowing within-person variability. They developed a nine-item questionnaire to describe their personal relationship with their mother, father, romantic partner, and best friends. Participants self-reported their degree of agreement and disagreement with each item for the specific close relationship using a seven-point scale. Six out of nine items were attachment-related avoidance, and three were attachment-related anxiety. Two of the attachment-related anxiety items were reverse keyed [15,16]. The adolescent AS was categorized based on the degree of anxiety and avoidance into: secure AS (low anxiety score [<4], and low avoidant score [<4]), dismissing AS (low anxiety score [<4], and high avoidant score [≥4]), preoccupied AS (high anxiety score [≥4], and low avoidant score [<4]), and fearful AS (high anxiety score [≥4], and high avoidant score [≥4]) [15]. A total of 31 adolescents completed the attachment-style questionnaire, and their AS was categorized as secure (7 adolescents), preoccupied (11 adolescents), dismissing (1 adolescent), and fearful (12 adolescents), as illustrated in Figure 1.

#### 2.4.2. Horse Heart Rate

Horses were equipped with heart rate (HR) monitors (Polar RS800, Polar Electro Canada, Lachine, QC, Canada) 20 min before the adolescents arrived to record horse HR during baseline, and during the grooming and riding components. Heart rate monitors consisted of an electrode strap, a transmitter, and a receiver. The electrode strap was an elastic surcingle specifically designed for horses, with built-in electrodes moistened with water and placed around the horse’s girth. The HR transmitter was attached to the surcingle, and the HR receiver was attached to the halter or bridle. The recording of horse HR started five minutes before adolescents arrived, and HR monitors recorded horse HR continuously every five seconds until the end of the arena component. These 5-s intervals were synchronized with the behavioural observation intervals. Heart rate baseline was calculated for each horse as the average HR two minutes before adolescents arrived.

#### 2.4.3. Horse Behaviour

The occurrence of affiliative, avoidance, and oral behaviours were recorded using interval one-zero sampling every five seconds for two-minute windows via live observations. Four observations windows were recorded per adolescent-horse dyad each session: two during the grooming and two during the riding component. Observation windows were spaced at least 10 min apart. The behavioural response of the horse toward the adolescent included *affiliative* (horse nudging adolescent, licking adolescent, moving toward adolescent, following adolescent’s requests), *avoidance* (horse shying, bolting, kicking, stomping, refusing to move, biting adolescent, pushing away adolescent, moving away from adolescent, moving in a speed or direction not asked for by adolescent, swishing tail, tossing head), and *oral behaviours* (horse licking, biting, teeth grinding, lip licking, chomping, gaping, tongue lolling). The occurrence of affiliative and avoidance behaviours were mutually exclusive, but not for oral behaviours. The frequency of affiliative, avoidance, and oral behaviours was calculated as the number of occurrences of that behavioural category per minute (i.e., counts/min). Ten trained volunteers assisted with the live observations of behaviours, and Kappa values of inter-rater agreement were acceptable for the three categories (ĸ = 0.86–1.0, *p* < 0.001). The riding gait of the horse was also recorded every five seconds as backing up, standing, or walking (trotting was not observed). 

### 2.5. Statistical Analyses

Behavioural and physiological data were analyzed using Generalized Linear Mixed Models with Proc Glimmix in SAS Ver. 9.4 (SAS Institute, Cary, NC, USA). Raw data are available in Supplementary Table S1. Data were separately analyzed by grooming and riding. During grooming, the AS of both adolescents were pooled together in the analyses because both adolescents interacted simultaneously with the horse. Differences associated with p-values lower than 0.05 were considered to be statistically significant.

The attachment style, week of the program, and its interaction were included as fixed factors in the models of grooming and riding. Riding gait was also added as a fixed factor into the analyses of the riding model, and baseline HR was included as a covariate in the analyses of horse HR response. Horse, adolescent, and gender were included as random factors, and observer was included in the analyses of the behavioural data as a random factor. The week of the program was fit into a repeated measure structure with the pair of adolescents (during grooming) or adolescent (during riding) as the subject. The p-values of the pairwise comparisons were adjusted for multiple comparisons using the Tukey test. Model assumptions were assessed using a scatterplot of studentized residuals, linear predictor for linearity, and a Shapiro–Wilk test for normality. The Gaussian distribution was used as the default distribution; however, data from the riding session were fit into logistic regression to meet normality and linearity criteria. The mean estimates of the riding session were back-transformed.

## 3. Results

The effect of the dismissing AS could not be analyzed statistically and was removed from the dataset because only one adolescent was associated with this AS (Figure 1). For the remaining adolescents, since both adolescents remained together by the horse during grooming, the following pairs of AS were observed: fearful and fearful (three pairs), preoccupied and fearful (three pairs), secure and fearful (three pairs), preoccupied and preoccupied (four pairs), and secure and preoccupied (three pairs). 

### 3.1. Grooming

**Figure 2:**
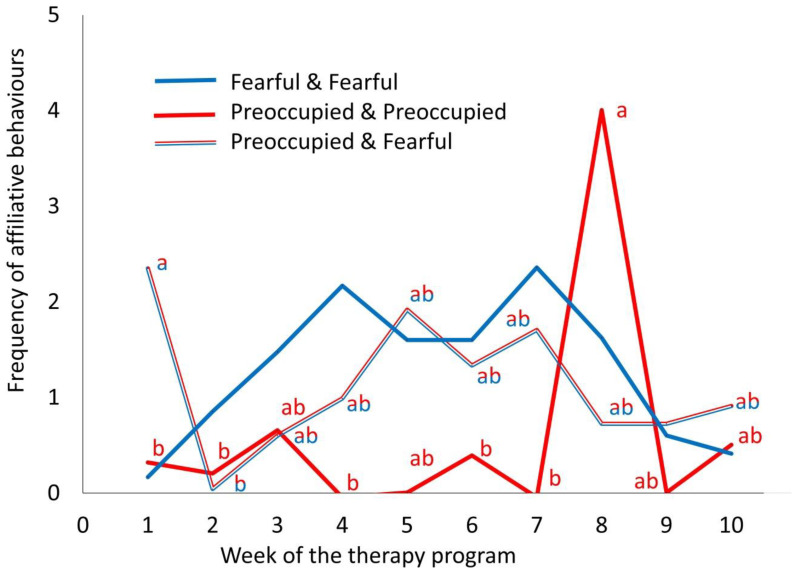
The effect of the human attachment style of 32 at-risk adolescents on the performance of affiliative behaviours by the therapy horses (n = 9) during the grooming component of a 10-week equine-facilitated learning program (mean ± SE). The blue line represents the pairs of adolescents exhibiting a fearful attachment style, the red line represents the pairs of adolescents exhibiting a preoccupied attachment style, and the red and blue line represents the pairs of adolescents with one showing a fearful attachment style and the other a preoccupied attachment style. Different letters indicate significant mean differences (*p* < 0.05) within attachment style pairings across week of the program. Differences over time were not significant for the behaviour of horses with both adolescents with a fearful attachment style.

The human AS of the adolescents affected the frequency of the affiliative behaviours of the horses depending on week during grooming (Figure 2; F_36,349_ = 1.96, *p* < 0.001), without an effect on horse heart rate (F_10,36_ = 1.05, *p* = 0.42) or the frequency of avoidance (F_10,36_ = 1.41, *p* = 0.21) and oral behaviours (F_10,36_ = 1.09, *p* = 0.39). Figure 2 illustrates that therapy horses with both adolescents with a fearful AS engaged in affiliative behaviours more consistently throughout consecutive sessions compared to those with both adolescents with a preoccupied AS. Overall, horses tended to show a higher frequency of affiliative behaviours toward the pair of adolescents in which both had a fearful AS (1.3 ± 0.2 counts per min) compared to those in which both had preoccupied AS (0.6 ± 0.2 counts per min; t_36_ = 2.60, *p* = 0.06) or secure and fearful AS (0.6 ± 0.1 counts per min; t_36_ = 2.91, *p* = 0.031). The average frequency of avoidance and oral behaviours was 0.9 ± 0.4 and 1.0 ± 0.4 counts per min, respectively. The average horse HR was 44.1 ± 1.6 beats per min, and the baseline HR had an effect on the horse HR during grooming (i.e., horses with a lower baseline HR also exhibited a lower HR during grooming; F_1,349_ = 34.3, *p* < 0.001). 

### 3.2. Riding

**Figure 3:**
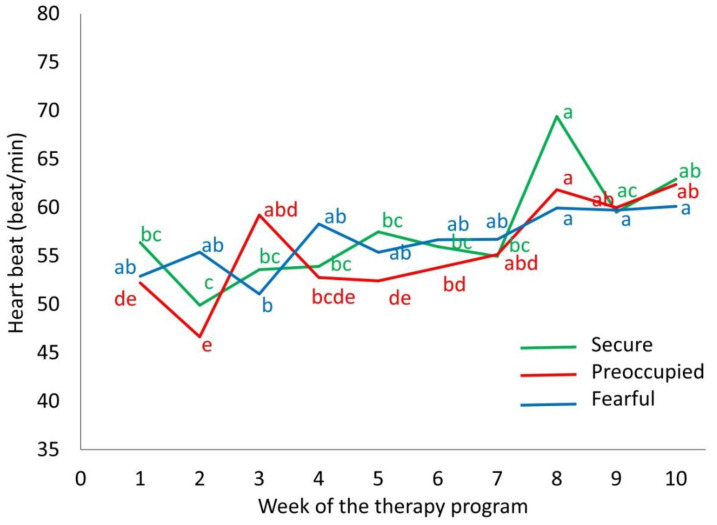
The effect of the human attachment style of 32 at-risk adolescents on the mean heart rate of therapy horses (n = 9) during the riding component of a 10-week equine-facilitated learning program. The green, red, and blue lines refer to adolescents with a secure, preoccupied, or fearful attachment style, correspondingly. Different letters (a–e) refer to significant mean differences in the heart rate of horses ridden by adolescents within the same attachment style across week of the program (*p* < 0.05).

**Figure 4:**
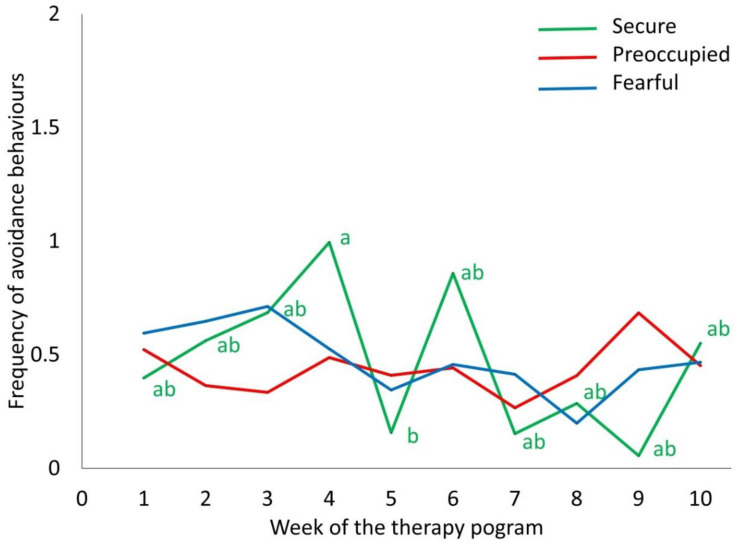
The effect of the human attachment style of 32 at-risk adolescents on the frequency of avoidance behaviours by therapy horses (n = 9) during the riding component of a 10-week equine-facilitated learning program (mean). The green, red, and blue lines refer to adolescents with a secure, preoccupied, or fearful attachment style, correspondingly. Different letters refer to significant mean differences in the behaviour of therapy horses ridden by adolescents with a secure attachment style across the week of the program (*p* < 0.05). Differences over time were not significant for the behaviour of horses ridden by adolescents with an insecure attachment style (preoccupied and fearful).

The human AS of the adolescents affected horse heart rate (F_18,279_ = 3.24, *p* < 0.001) and the frequency of avoidance behaviours (F_18,279_ = 2.01, *p* = 0.009) of the horses while riding, depending on the week. Average horse heart rate increased from 49.8 beats per min at week 2 to 60.0 beats per min at week 10 (t_9_ = 7.25, *p* = 0.001), but this increase was steadier in horses ridden by adolescents with a fearful AS, compared to those ridden by adolescents with a preoccupied or secure AS (Figure 3). Horses ridden by adolescents with a fearful or preoccupied AS showed a constant frequency of avoidance behaviours while riding (0.9 counts per min), whereas those ridden by adolescents with a secure AS showed a significantly irregular pattern of avoidance behaviours (Figure 4). In horses ridden by adolescents with a fearful AS, the frequency of avoidance behaviours toward adolescents decreased over time (Figure 4; t_279_ = 3.77, *p* = 0.05). In horses ridden by adolescents with a secure AS, the frequency of avoidance behaviours toward adolescents was the highest at week 4 and tended to be higher than at week 7 (Figure 4; t_279_ = 3.71, *p* = 0.06) and week 9 (Figure 4; t_279_ = 3.63, *p* = 0.08). 

The duration of the EFL program affected horse behaviour (frequency of affiliative: F_9,9_ = 12.41, *p* < 0.001; and oral behaviours: F_9,9_ = 4.15, *p* = 0.023) while riding. Horses performed exponentially less affiliative behaviours throughout the duration of the EFL program (from 0.5 counts per minute at week 1 to 0.0 counts per minute at week 10; t_9_ = 7.62, *p* < 0.001), regardless of adolescents’ AS. Horses also performed less oral behaviours during the first half (0.2 counts per minute during weeks 1 to 4) compared to the second half (0.4 counts per minute during weeks 5 to 10) of the EFL program, regardless of adolescent AS. 

Riding gait affected the physiological (F_2,722_ = 8.32, *p* < 0.001) and behavioural response of the horses (frequency of affiliative (F_2,785_ = 3.02, *p* = 0.049), avoidance (F_2,786_ = 28.18, *p* < 0.001), and oral behaviours (F_2,785_ = 9.61, *p* < 0.001)). Even accounting for physical exertion, compared to standing still, horses had higher heart rates (58.2 vs. 55.5 beats per min; t_722_ = 3.78, *p* < 0.001), and higher frequency of avoidance behaviours (1.1 vs. 0.6 counts per min; t_786_ = 6.39, *p* < 0.001) while walking, and had a lower frequency of affiliative (0.0 vs. 0.1 counts per min; t_785_ = 2.46, *p* = 0.038), avoidance (0.4 vs. 0.7 counts per min; t_786_ = 2.68, *p* = 0.020), and oral behaviours (0.2 vs. 0.7 counts per min; t_785_ = 4.13, *p* < 0.001) while backing up. 

## 4. Discussion

The physiological and behavioural response of therapy horses was hypothesized to differ among adolescents with different AS according to previous research [3,9,10,23]. The results from this research partially corroborate our hypothesis, as the effect of the adolescent’s AS on horse response varied depending on the component of the EFL program. The AS of at-risk adolescents affected the frequency of affiliative behaviours during grooming and affected the horse HR and frequency of avoidance behaviours during riding. 

The physiological and behavioural response of therapy horses was more predictable when paired with adolescents with fearful or preoccupied AS compared to those with secure AS. These results indicate that therapy horses were sensitive to behavioural and/or emotional differences associated with the human AS of adolescents participating in an EFL program. Previous results indicated that the physiological and behavioural response of horses can be affected by human traits [9,10,26,27]. Merkies and colleagues [9] reported that horses had a lower HR when in the presence of humans that ranked themselves as fearful around horses. Indeed, horses showed a lower stress response (slower gait and lower head position) when exposed to physically and psychologically stressed humans compared to calm humans [9]. Similar results were reported by Kaiser et al. [20] and Johnson et al. [3] during EAT. Kaiser and colleagues [20] observed that therapy horses during therapeutic riding showed a lower frequency of stress-related behaviours when ridden by at-risk children and by those who are physically and psychologically handicapped than when ridden by advanced riders. Johnson and colleagues [3] observed that therapy horses during EAT scored lower in stress-related behaviours when ridden by adult humans with severe physical, emotional, and mental disabilities than when ridden by experienced riders. Chamove and colleagues [28] indicated that human confidence was negatively correlated with horse resistance (i.e., reluctant to move and/or moving away). These results suggest that human attitude toward horses can impact horse behaviour, and the behavioural response of horses can differ between experienced riders and at-risk humans during EAA. Merkies and colleagues [10] did not observe a change in the behavioural response of horses in the presence of mentally traumatized vs. control humans, but the horse HR increased during and after exposure to the mentally traumatized humans. Control humans in that study were professionally trained to simulate the physical body language of mentally traumatized humans, leading to the conclusion that the behavioural response of therapy horses may rely on human physical traits rather than affective traits [10]. Similarly, the high variability observed in our study between the heart rate and the frequency of avoidance behaviours of therapy horses with secure AS adolescents did not correspond with each other. These results might suggest that the mechanisms behind the physiological and behavioural responses of therapy horses may involve different pathways. In support of this, Squibb and colleagues [29] determined that there was no correlation between proactive behaviour and horse HR when the horses were asked to perform stressful tasks (walk over a tarp or through streamers). Further, Ijichi and colleagues [13] described no differences in the behavioural or physiological responses of horses led by a familiar vs. unfamiliar handler through two novel object tests. Human traits can, therefore, impact horse response, although the casual factors involved in the human–horse interaction are unclear. Emotional intelligence (EI: i.e., an individual’s capability of emotional self-awareness, and ability to recognize the emotional awareness of others and respond accordingly) has been suggested to underline attachment and emotional bonding in human–animal interactions [6]. Indeed, the degree of EI in humans can influence the quality and effectiveness of human–animal interaction [6,20]. Individuals with a secure AS are associated with high EI scores, whereas those with insecure AS are associated with low EI [30]. For this reason, differences in EI can potentially explain the predictability of the physiological and behavioural responses of therapy horses described in our study. However, the adolescent HR, attitude, EI, or behaviour toward their horse was not recorded in the current study in order to not interfere with the ongoing EAA. 

The variability of the horse response toward at-risk adolescents with secure AS can raise safety concerns around horses [22] and may underlie negative consequences for horse welfare [21,23]. Elevated heart rate and the performance of avoidant and agonistic behaviours toward humans is associated with distress, fear, anxiety, and discomfort under stressful conditions [21,23]. In our study, the horse HR was lower than 75 beats per min while riding (at the walk), and the avoidance and agonistic behaviours observed were low intensity (e.g., ears back, tossing head, swishing tail, reluctance to obey rider’s command). Therefore, our results do not suggest that therapy horses in this EFL program showed signs of physiological or behavioural distress, in line with previous research [3,20,27,31,32]. Therapy horses in our study certainly showed affiliative behaviours toward adolescents during grooming and the first riding sessions. Physical contact, such as petting and scratching, and other affiliative behaviours by humans, can reduce fear responses and promote positive affective states in horses [6,7,8,9,10,11,12,13,14,15,16,17,18,19,20,21], and vice versa. Physical contact, social interaction, and bonding during EFL can lead to an emotional connection between the adolescent and the horse, facilitating the development of functional relationships in insecure adolescents [2]. Human–horse interaction includes a whole array of social interactions, including agonistic, affiliative, cooperative, and avoidance behaviours [5,6,7,8,9,10,11,12,13,14,15,16,17,18,19,20,21]. Despite the negative connotation of avoidance and aggressive behaviours for horse welfare, the behavioural feedback of therapy horses is a direct reaction to human body language, behaviour, and/or physical attributes during EAT and EFL programs [2,5]. This behavioural response of therapy horses is important to raise awareness about participants’ attitudes and emotional issues and offers educational opportunities to achieve learning milestones in EAT and EFL sessions [2,5]. As previous research has suggested (e.g., [2]), our results indicate that the behavioural flexibility of the horse’s response is context-dependent and varies across sessions. As well as this, horses can habituate to acute stress due to exposure to unfamiliar and unpredictable humans during EAA sessions [10]. Habituation could explain the lack of differences in the horse HR during grooming, but, at least in this pilot study, the behavioural response was sensitive to adolescents’ AS and provides a means to assess therapy horse welfare [23]. Aside from the therapeutic purpose of EAT and EFL sessions, understanding the welfare of therapy horses is equally important during therapy sessions to safeguard positive horse welfare and the participant’s safety [3,22]. 

### Limitations of the Study

The results of this study provide information regarding the human–horse interaction, particularly about the response of therapy horses toward at-risk adolescents with varying human AS under EAA conditions. However, one limitation associated with the use of the ECR-RS in this study, and measuring AS in general [15,16], is the difficulty of categorizing human AS on the secure end (in between the secure and insecure edge). Efforts to analyze the effect of the adolescent AS on the physiological and behavioural responses of therapy horses quantitatively (e.g., continuously) resulted in the overdispersion of the cross-products of the attachment-related anxiety and avoidance data. Quantitative analyses were therefore excluded in this study, but this limitation should be acknowledged in future research measuring or assessing the effect of AS. Another limitation of this study is that we did not measure the AS of adolescents toward their therapy horses or animals in general. The ECS-RS used in this research [16] measured adolescents’ AS among four different close relationships (with their mother, father, romantic partner, and best friends), and there might be differentiation or heterogeneous AS in a variety of relationship contexts (e.g., humans vs. horses). Adolescents with insecure AS toward humans might be likely to behave accordingly toward animals [17,18]. Indeed, Kaiser et al. [20] indicated that at-risk children may see horses as tools rather than creating an emotional connection. Alternatively, insecure adolescents with dysfunctional relationships with humans can express their feelings more willingly to horses than to a human therapist, and therapy horses can provide safe and non-judgmental companionship, enabling anxious and avoidant adolescents to connect with them physically and emotionally [2,4,5]. For this reason, a positive client–horse interaction is key for the participation rate and the beneficial effect of EAA [1,2,4]. However, other factors such as individual differences (in humans and animals), EFL program planning and setting, duration of the sessions, and modality (individually vs. group) can alter the outcomes of the EAT and EFL program [1]. Identifying the factors and management strategies that reduce fear responses in horses (e.g., human attitude and AS toward horses, emotional intelligence, human HR and behaviour) can improve the human–animal relationship and address safety concerns around horses [6,22]. 

## 5. Conclusions

These results indicate that therapy horses did not exhibit physiological or behavioural distress during the ETL program, and the human attachment style (AS) of the at-risk humans with emotional and mental difficulties can influence the horse response during the human–horse interaction. The physiological and behavioural response of therapy horses was more predictable toward adolescents with a fearful or preoccupied AS versus those with a secure AS. These results indicate that a low-stress response of therapy horses toward participants in an EFL program with emotional and behavioural difficulties may be mediated by a human insecure attachment style. This provides novel insights into the human–horse interaction under EAA settings; however, the underlying mechanisms behind the more predictable response of a horse toward highly anxious and highly avoidant adolescents are yet to be determined.

## Supplementary Materials

Table S1: raw data is available online at Mendeley Data, v1 http://dx.doi.org/10.17632/4yhc64z9n2.1.
